# Ripple coarsening on ion beam-eroded surfaces

**DOI:** 10.1186/1556-276X-9-439

**Published:** 2014-08-27

**Authors:** Marc Teichmann, Jan Lorbeer, Frank Frost, Bernd Rauschenbach

**Affiliations:** 1 Leibniz-Institut für Oberflächenmodifizierung (IOM), Permoserstr. 15, D-04318 Leipzig, Germany

**Keywords:** Ion beam patterning, Ripple coarsening, Silicon, Germanium, Fused silica, Sapphire

## Abstract

The temporal evolution of ripple pattern on Ge, Si, A*l*_2_*O*_3_, and Si*O*_2_ by low-energy ion beam erosion with Xe ^+^ ions is studied. The experiments focus on the ripple dynamics in a fluence range from 1.1 × 10^17^ cm^-2^ to 1.3 × 10^19^ cm^-2^ at ion incidence angles of 65° and 75° and ion energies of 600 and 1,200 eV. At low fluences a short-wavelength ripple structure emerges on the surface that is superimposed and later on dominated by long wavelength structures for increasing fluences. The coarsening of short wavelength ripples depends on the material system and angle of incidence. These observations are associated with the influence of reflected primary ions and gradient-dependent sputtering. The investigations reveal that coarsening of the pattern is a universal behavior for all investigated materials, just at the earliest accessible stage of surface evolution.

## Background

Topography engineering by ion beam erosion has attracted much interest for patterning [1-8] and surface smoothening [9,10] for several years. In the last decade, many investigations have focused on Si as a model material in order to explore the origin of ion beam patterning [7,11-15]. The first model that was able to describe formation of self-organized nanostructures is the erosion-based theory of Bradley and Harper (BH) [16]. The model explains the ripple formation process and orientation of ripples with respect to the ion beam direction by means of surface-curvature-dependent sputtering and temperature-dependent diffusion. Nevertheless, the model cannot explain several experimental findings. Especially a transition between a smooth and rippled surface at a certain angle of incidence is not explained. In addition to the BH model, Carter and Vishnyakov (CV) [17] suggested ballistic drift due to momentum transfer during ion bombardment as smoothening mechanism. In consequence, the ripple formation occurs if curvature-dependent sputtering can compensate the ballistic drift. Madi et al. [18] combined both approaches and modified them with a correction factor for high incidence angles. This model predicts a critical angle as starting point for patterning and explains smoothening for normal and near-normal incidence angles. Similar results have also been shown by the crater function theory of Norris et al. [19,20]. The moments of the crater function also consist of an erosive and a dominating redistributive part.

A key factor to address the applicability of different models is the dynamic behavior of pattern formation. In generally accepted models, two regimes are distinguished [21]. The first regime is the linear time regime of pattern formation where the wavelength of the pattern is constant. The crossover into the nonlinear regime begins if the wavelength grows with irradiation time. Alternatively, the regimes can be separated by the temporal evolution of the surface roughness which grows exponentially in the linear regime and has a power-law behavior in the nonlinear regime. Furthermore, the total ion fluence is a critical parameter that controls the structure size and regularity of the ripple pattern.

Former work revealed that coarsening by reflected ions played a crucial role for surface evolution. The mechanism has been introduced by Hauffe [22] and is considered to account for the ripple coarsening that was observed on Si*O*_2_[23] and Ge [24]. The aim of this work is to present a comprehensive study of low-energy ion beam erosion, especially of the role of reflection of primary ions for different technical important substrate materials with Xe gas ions. In particular Si is used as reference material that is well investigated in the last decade. As direct counterpart to Si, Ge is used, which has a similar sputter yield as Si but a higher fraction of reflected ions due to its higher target mass. The oxidic pendant Si*O*_2_ (fused silica) of Si is analyzed as well as A*l*_2_*O*_3_ (sapphire), which have comparable reflection coefficients. However, A*l*_2_*O*_3_ has a lower sputter yield than Si*O*_2_ because of its higher surface-binding energy. As sputtering gas, Xe was used for which distinct pattern formation takes place on all target materials. As incidence angles, 65° and 75° were chosen, where ripple formation takes place and the sputter yield is close by its maximum.

## Methods

The samples used were commercially available polished Ge(100) (initial root mean square (RMS) roughness $R_{q}^{0}\approx 0.5\;\text{nm}$), Si(100) $\left(R_{q}^{0}\approx 0.2\;\text{nm}\right)$, A*l*_2_*O*_3_$\left(1\bar{1}02\right)$$\left(R_{q}^{0}\approx 0.1\;\text{nm}\right)$ and amorphous Si*O*_2_$\left(R_{q}^{0}\approx 0.5\;\text{nm}\right)$ substrate pieces. These samples were mounted on a water-cooled substrate holder in a high vacuum chamber with a base pressure of 10^-6^ mbar, which can be tilted from 0° (corresponding to normal ion incidence) up to 90° with respect to the axis of the ion beam source. The cooling ensures a substrate temperature below 80°C. Furthermore, the sample holder is equipped with a silicon shielding in order to prevent metallic contaminations that affect the evolving structures on Si [14,25] as well as on Ge [24,26]. This non co-deposition setup also prevents secondary collisions of scattered gas ions and redeposition of sputtered silicon atoms [27]. For the experiments, a homebuilt Kaufman-type ion source is used. Furthermore, the source is equipped with two 190-mm grids with a reduced aperture of 100 mm. Hence, no metallic contaminations could be detected with Rutherford backscattering spectrometry (RBS) as well as with X-ray photoelectron spectroscopy (XPS) measurements. The Xe ^+^ ion current density was kept constant at 300 *μ*A/cm^2^ during the experiments which results in an ion flux of *J *= 1.87 × 10^15^ cm^-2^ s^-1^ in a plane perpendicular to the ion beam. The samples were irradiated for durations from 1 up to 120 min corresponding to a fluence range from 1.12 × 10^17^ cm^-2^ to 1.35 × 10^19^ cm^-2^. An ion energy of 600 and 1,200 eV was used. The surface topography was analyzed by scanning atomic force microscopy (AFM) operating in TappingMode™ or ScanAsyst™ mode [28]. The measurements were performed in air using silicon nitride cantilever with Si tips with a nominal tip radius smaller than 5 nm (TappingMode™; Bruker Corporation, Billerica, MA, USA) or silicon nitride cantilevers with nominal tip radius of 2 nm (ScanAsyst™; Bruker Corporation). Routinely, each sample was analyzed with a scan size of 2 or 4 *μ*m and 10 *μ*m and a resolution of at least 1,024 × 1,024 pixels. The AFM data were analyzed with SPIP™ [29] software and a custom-programmed MATLAB^®^; (Mathworks Inc., Natick, MA, USA) routine for calculation of the power spectral density (PSD) functions following an approach of Duparré et al. [30]. The wavelengths of the ripple structures are determined from the corresponding PSD curve. For a given sample (sample size typically 10 mm × 10 mm), the wavelength determined at different positions across the sample is extremely small. Also, the fluctuation of the corresponding RMS surface roughness is negligible small. Therefore, no error bars are indicated in the graphs as they are typically smaller than the symbol size. However, it must be noted that the experimental reproducibility between different etching series is in the range ≤5%. For surface gradient angle calculation, SPIP™ was used again and the gradient angles are calculated in ion beam projection direction. The gradient histograms have been computed from the x-gradient images.

## Results

**Figure 1:**
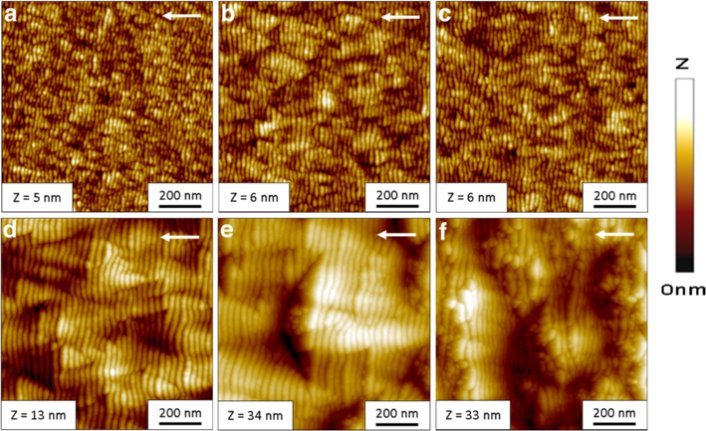
**AFM images of Xe **^**+**^** ion beam-eroded fused silica surfaces for*E***_**ion**_**= 600 eV,*j***_**ion**_**= 300 *μ*A/cm**^**2**^** and ion incidence angle of *α***_***ion***_***= 65 *° for increasing fluences. (a) ***ϕ* = 2.25 × 10^17^ cm^-2^, **(b)** 5.62 × 10^17^ cm^-2^, **(c)** 1.24 × 10^18^ cm^-2^, **(d)** 3.37 × 10^18^ cm^-2^, **(e)** 6.74 × 10^18^ cm^-2^, and **(f)** 1.35 × 10^19^cm^-2^. The image size is 1×1 *μ*m^2^. The different height scales of the images are specified in each image. The white arrow indicates the projection of the ion beam direction.

**Figure 2:**
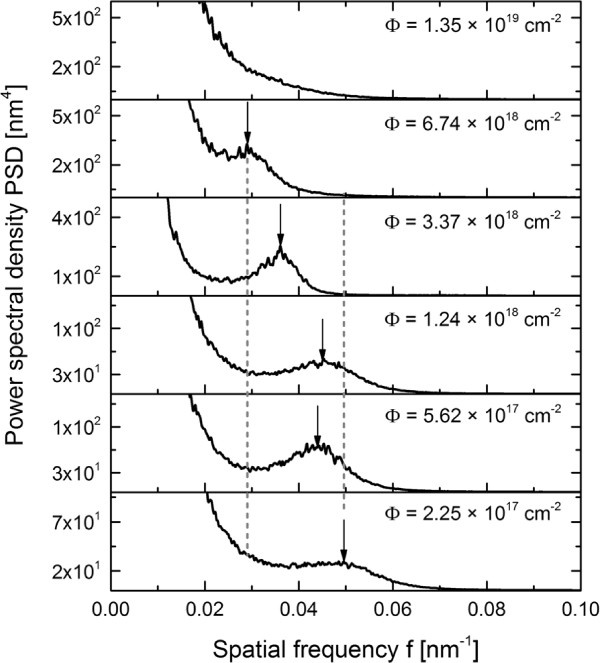
**Calculated PSD of images of Xe **^**+**^** ion beam eroded fused silica surfaces for fluences.** From *Φ* = 2.25 × 10^17^ cm^-2^ up to *Φ* = 1.35 × 10^19^ cm^-2^, *E*_ion_ = 600 eV, *j*_ion_ = 300 *μ*A/cm^2^, ion incidence angle of *α* = 65°. The dotted vertical lines mark the shortest and longest ripple wavelength, respectively. The arrows highlight the wavelength at the corresponding fluence.

The experiments presented here focus on the time evolution of the surface pattern. In the first case, an incidence angle of 65° was chosen where distinct ripple formation occurs for all investigated material systems. Some characteristic AFM images of the ripple pattern formed on fused silica for this case are shown in Figure 1^a^. The total fluence is increasing from Figure 1a,b,c,d,e,f. The amplitude and regularity of the ripple pattern increase with erosion time until a total fluence of 3.4 × 10^18^ cm^-2^ (Figure 1d) is reached. In the early stages, an irregular ripple pattern with a ripple wavelength of about 20 nm and RMS of 0.85 nm is visible. If the total fluence is increased already, triangular hillocks and depression are evolving in between the pattern. This can be seen for the fluence of 5.6 × 10^17^ cm^-2^ (Figure 1b) and is more pronounced for longer erosion times. Around the crests the ripples are bent and the ripple wavelength is slightly changed caused by small variations of the local ion incidence angle. The increase of the wavelength and ripple regularity can be clearly seen in the PSD curves in Figure 2. The peak position shifts from *f*≈0.05 nm^-1^ at a fluence of 2.3 × 10^17^ cm^-2^ to lower spatial frequency of *f *≈ 0.03 nm^-1^ at a fluence of 6.7 × 10^18^ cm^-2^ which corresponds to larger wavelengths. This is illustrated by means of the dotted lines in Figure 2, which show the smallest and largest spatial frequency of the corresponding ripple pattern. Furthermore, the width of the peak reduces with time indicating a higher regularity. In the low-frequency range (≤0.03 nm^-1^), the amplitude of the PSD curves strongly grows. This corresponds to a higher surface roughness related to long-wavelength surface disturbances. If the erosion time is increased further (Figure 1e), larger faceted structures are emerging on the surface. The analysis of the surface gradients shows a certain slope of the facets, namely, 13° on the downstream and -11° on the upstream side which corresponds to a local angle of incidence of 78° and 54°. Around these structures, a clear ripple pattern with a mean wavelength of about 34 nm can be recognized. For the longest erosion time (Figure 1f), the faceted structures grow in size and begin to overlap, accompanied by a higher RMS roughness of about 7.35 nm. Furthermore, the size of the downstream facets increases and their angle towards the global surface is 13°.

**Figure 3:**
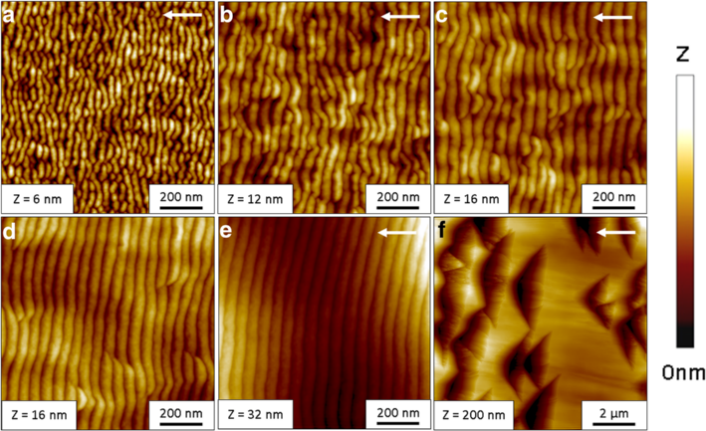
**AFM images of Xe **^**+**^** ion beam-eroded Si surfaces for *E***_**ion**_**= 1,200 eV,*j***_**ion**_**= 300 *μ*A/cm**^**2**^** and ion incidence angle of *α***_**ion**_**= 75° for increasing fluences. (a) ***ϕ* = 1.12 × 10^17^ cm^-2^, **(b)** 5.62 × 10^17^ cm^-2^, **(c)** 1.12×10^18^ cm^-2^, **(d)** 3.37 × 10^18^ cm^-2^, and **(e)** 1.35 × 10^19^ cm^-2^. The image size is 1 × 1 *μ*m^2^ (a to e) and 10 × 10 *μ*m^2^ for the zooming of the 1.35 × 10^19^ cm^-2^ sample **(f)**. The different height scales of the images are specified in each image. The white arrow indicates the projection of the ion beam direction.

**Figure 4:**
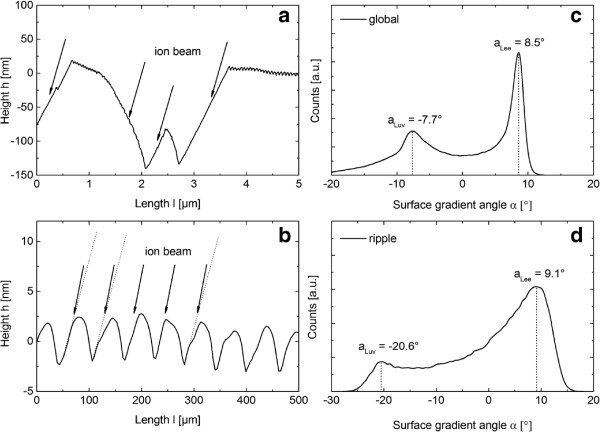
**Height profiles and distribution of the surface gradient angles of Figure **3**f.** The profiles are along the triangular depressions **(a)** and in region where only ripples exist **(b)**. The arrows indicate the direction of the ion beam incidence. The angular distribution are evaluated for the entire AFM image **(c)** and in the region where only ripples occur **(d)**. *α *< 0° correspond to the upstream side and *α *> 0° to the downstream side of the facets.

The second set of experiments were carried out at 1,200 eV and an ion incidence angle of 75°, and a regime where significant faceting appears beside ripple formation. The fluence was varied from 1.12 × 10^17^ cm^-2^ to 1.35 × 10^19^ cm^-2^. Exemplarily, the AFM images of Si are shown in Figure 3. For the shortest erosion time (Figure 3a), an irregular ripple pattern with a mean wavelength of about 37 nm evolves on the surface. For longer durations of irradiation, the pattern becomes more regular, as well as the ripple wavelength and RMS roughness of the surface increase. This behavior is similar to the observations of Si*O*_2_. In particular, in Figure 3b a wavelength of 44 nm and RMS value of 1.51 nm are observed. In Figure 3c,d the wavelength increases further from 47 to 53 nm, respectively. In between this highly regular pattern, large triangular depressions emerge on the surface similar in their appearance to the ones on Si*O*_2_ (Figures 1e and 3f). In contrast to Si*O*_2_, these structures are larger on Si. Additionally, the amplitude and the wavelength of the ripples are higher on Si. The faceted structures have a well-defined orientation towards the global surface normal. This can be seen in the height profile along these triangular depressions (Figure 4a) and the distribution of the facet angles (Figure 4c). They form an angle of about 8.5° towards the global surface on the downstream side which corresponds to a local angle of incidence of 83.5°. The local angle of incidence on the upstream side is approximately 67°. Furthermore, the ripple structure between the depressions is also faceted (Figure 4b and 4d). The angle of the downstream facet is similar whereas the upstream side strongly differs. Based on the calculated local ion incidence angles it is evident that shadowing is not relevant. This is also seen from the height profiles shown in Figure 4a,b.

**Figure 5:**
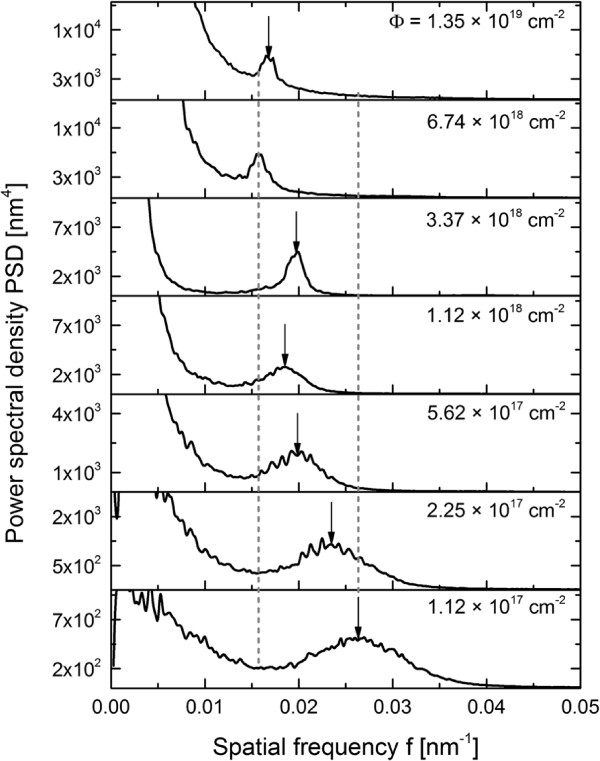
**Calculated PSD of images of Xe **^**+**^** ion beam-eroded Si surfaces for fluences.** Fluences are from *Φ *= 1.12 × 10^17^ cm^-2^ up to *Φ *= 1.35 × 10^19^ cm^-2^, *E*_ion_= 1,200 eV, *j*_ion_= 300 *μ*A/cm^2^, ion incidence angle of *α *= 75°. The dotted vertical lines mark the shortest and longest ripple wavelength, respectively. The arrows highlight the wavelength at the corresponding fluence.

The development of the Si surface depending on fluence is summarized in Figure 5. It shows a stack of PSD curves calculated from the 4 × 4 *μ*m^2^ images. Thereby, the general trend of the wavelength as well as the roughness evolution is illustrated. The dotted lines mark the shortest and longest wavelength. Basically, the same behavior as for Si*O*_2_ is observed for Si, i.e., wavelength and ripple ordering increases with erosion time.

**Figure 6:**
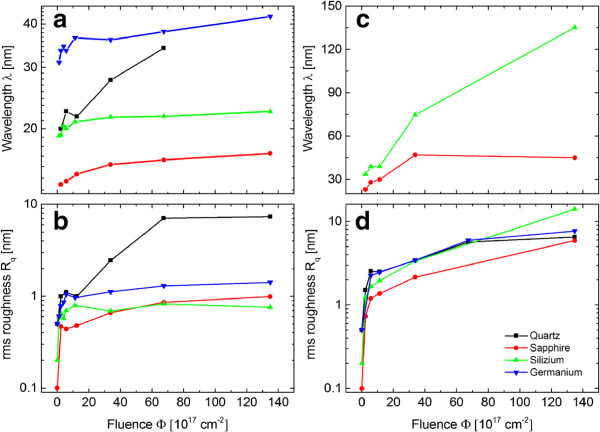
**Structure wavelength and RMS roughness as a function of fluence for Xe **^**+**^** irradiated samples for fluences.** Fluences are from *Φ* = 1.12 × 10^17^ cm^-2^ up to *Φ* = 1.35 × 10^19^ cm^-2^. *E*_ion_ = 600 eV, ion incidence angle of *α* = 65° **(a, b)** and *α* = 75° **(c, d)**.

A summary of the results done at 600 eV is exemplarily given in Figure 6 for all investigated materials. It illustrates the general trends in the temporal evolution of the structure wavelength and the surface roughness for various materials. In Figure 6a,c the structural wavelength is shown as a function of fluence for 65° and 75°. The wavelength and roughness increase nonlinearly with fluence. A strong growth is observed for Ge and Si*O*_2_, e.g., the ripple wavelength increases from the lowest to the highest fluence by 70% in case of Si*O*_2_. In contrast, the ripple wavelength rises for Si and A*l*_2_*O*_3_ only about 20% and 25% for the given fluence. In Figure 6c no curves are plotted for Ge and Si*O*_2_ because the surface is strongly faceted and therefore no wavelength can be obtained from these images. It is evident that ripple coarsening is much more pronounced for Si and A*l*_2_*O*_3_ at 75° compared to 65°. Figure 6b,d illustrates the evolution of the surface roughness with increasing fluence. Especially, a strong growth of the roughness is observed for small fluences.

## Discussion

**Figure 7:**
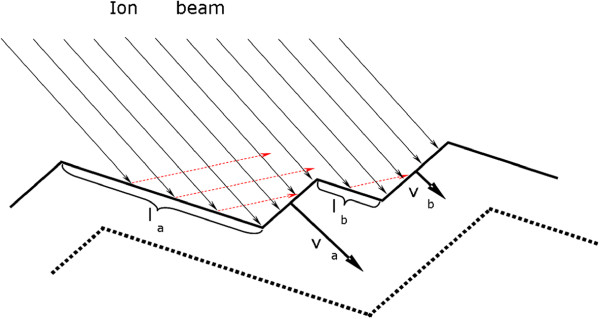
**Sketch to illustrate coarsening by reflected ions.** More ions are reflected from the larger surface element *l*_a_ than from the smaller surface element *l*_b_. Therefore, the local erosion velocity *v*_a_ is enhanced in comparison to *v*_b_.

**Figure 8:**
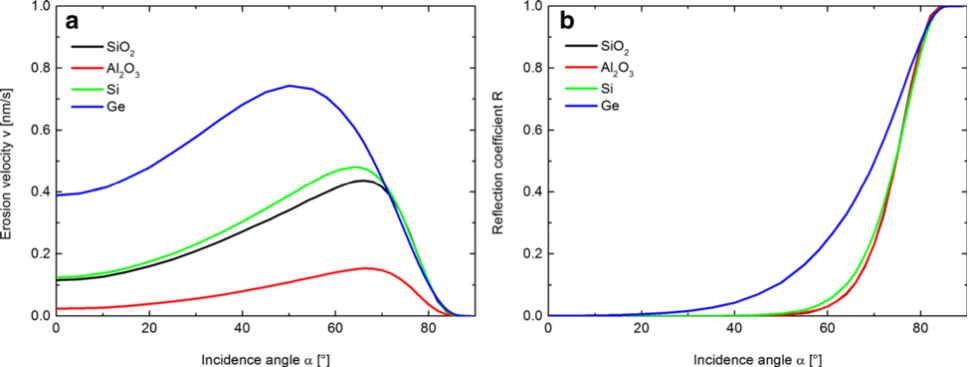
**Erosion velocity *v * and reflection coefficients *R *.** Erosion velocity *v ***(a)** and reflection coefficients *R ***(b)** as function of incidence angle for different material systems with Xe at ion energy *E*_ion_ = 600 eV based on TRIM.SP calculations [31]. The erosion velocity *v* was calculated using an ion current density of 300 *μ*A/cm^2^, the individual, angular dependent sputter yields (from TRIM.SP), and the atomic densities of the materials.

For both angles of incidence, a clear evidence of coarsening and faceting of surface pattern is found. In the experiments a continuous increase of the structure wavelength as well as surface roughness is observed for higher fluence (Figure 6). This behavior is independent of the material and ion energy but differently developed over the investigated fluence range. The coarsening of the structures is caused by reflected primary ions. These ions are directed towards adjoining structures, where the local erosion rate is enhanced due to the additional ion flux (see Figure 7). This mechanism, proposed by Hauffe [22], explains why smaller features close to larger ones vanish and the surface coarsens. This effect is strongly enhanced at larger incidence angles because of the higher fraction of reflected primary ions. For comparison, the reflectivity of Xe ions with an energy of *E*_ion_ = 600 eV that hit Si is *R*≈0.12 and *R*≈0.53 for an ion incidence angle of *α*_ion_ = 65° and *α*_ion_ = 75° (Figure 8b), respectively. Based on TRIM.SP calculations [31], one can state that the reflectivity evidently increases with ion incidence angle but also with increasing target mass, decreasing projectile mass, and decreasing ion energy. Therefore, a stronger coarsening is observed in experiments for 75° in comparison to 65° (Figure 6). An alternative coarsening mechanism, included in continuum models for mound formation in epitaxial growth processes [32] or important for ion erosion of single crystalline metal surfaces [33], is attributed to a Schwoebel barrier for interlayer diffusion [34-37]. However, this mechanism is not relevant for the observed coarsening because thermal surface diffusion can be neglected and, furthermore, all investigated materials are amorphous (Si*O*_2_) or amorphized during ion erosion (Si, Ge, A*l*_2_*O*_3_).

Another observable tendency in the evolution of the structures is the formation of faceted structures with a distinct angle towards the global surface normal. Such a process is potentially explained by gradient-dependent sputtering. In accord with Nobes et al. [38] and Johnson [39], stable facets evolve on the surface with local angle of incidence of 0°, 90° and the angle of incidence where the erosion rate is maximal. However, these angles are not exactly found in the evaluation of the surface gradients because the process is superimposed by smoothening mechanisms and the influence of reflected ions. Moreover, depending on the slope of the erosion velocity curve (Figure 8a), one can make a point to the dynamic of the system: a greater slope of the curve causes a faster evolution of the surface.

In the linear stability regime, a constant wavelength is predicted with increasing fluence. This is usually described by linear differential equations until a critical value is reached. For higher fluences the system has to be described in a nonlinear regime as the approximations for a linear stability are not admissible anymore. This raises the question if for shorter erosion times linear behavior can be observed. Castro et al. [21] have defined an intrinsic time scale ensuing from their experiments which describes the transition from linear to nonlinear regime. If the given calculation rule is applied to our experimental conditions, this leads to very small fluences. This fluence range is not accessible in our experimental setup. Nevertheless, the emerging ripple patterns in this fluence range have very small amplitude and regularity, which are of little relevance for technical applications. Finally, the resulting material removal is extremely low. Hence, the technological fluence range is inevitable in the nonlinear regime, and reflected ions as well as gradient-dependent sputtering have to be considered. However, the reflection of primary ions as a nonlocal process is beyond the current theoretical models. The only nonlocal process that is considered in current theoretical models is redeposition [40,41].

## Conclusions

Ripple dynamic is investigated on Ge, Si, A*l*_2_*O*_3_, and Si*O*_2_ by low-energy ion beam erosion for ion energies of 600 and 1,200 eV and ion incidence angles of 65° and 75°. The ion fluence was varied from 1.1 × 10^17^ cm^-2^ to 1.3 × 10^19^cm^-2^ for both angles of incidence. Coarsening of the emerging ripple pattern is observed independent of the material already for the smallest ion fluences. This coarsening behavior has been attributed to reflection of primary ions. The coarsening rate depends on the fraction of reflected primary ions and therefore depends on the specific material as well as on the angle of incidence. The so-called Hauffe mechanism necessitates that experiments and theories have to be considered in the nonlinear regime.

## Endnote

^a^ AFM images of other materials can be made available on request.

## Competing interests

The authors declare that they have no competing interests.

## Authors’ contributions

MT wrote the paper and performed irradiation experiments and other analysis. JL performed irradiation experiments. FF was responsible for the planning of the experiments, conducted AFM measurements, and helped during the manuscript preparation. BR contributed to the discussion of results and incorporated final corrections into the manuscript. All authors read and approved the final manuscript.

## Authors’ information

This research was conducted at the Leibniz Institute of Surface Modification (IOM) in Leipzig, Germany. The team is part of a collaborative research unit funded by the German Research Foundation (DFG) which is focused on the formation of self-organized nanostructures through low-energy ion beams (FOR 845). MT and JL are PhD students in two subprojects of research unit FOR 845. Dr. FF is the head of the group ‘Ion beam assisted technologies’ at the IOM and the project leader of two subprojects in research unit FOR 845. Prof. Dr. Dr. BR is the director of the IOM Leipzig as well as spokesperson of research unit FOR 845. He is also member of the curatorship for ‘Innovation and Science,’ member of the coordination board ‘Plasma Surface Technologies,’ member of ‘Leipziger Forschungsforum’ at the University Leipzig, member of the advisory board of the International Conference on Plasma Surface Engineering, member of the editorial board of *Journal of Materials*, member of the advisory board of the International Conference on Ion, Electron and Laser Physics, member of the Internal advisory committee of the Translational Centre for Regenerative Medicine, member of the scientific committee of the International Conference of Surface Modification of Materials, member of the editorial board ‘Dataset of Material Science,’ member of the editorial board of *Condensed Matter Physics* and member of the scientific committee ‘Nanomaterials: Applications and Properties.’
